# Supplementary material for: Discriminant Canonical Tool for Differential Biometric Characterization of Multivariety Endangered Hen Breeds
Source: Animals (Basel). 2021 Jul 26;11(8):2211. doi: 10.3390/ani11082211 (PMC8388411; doi:10.3390/ani11082211)
Supplement: Supplementary file 1 [file animals-11-02211-s001.zip › Supplementary Table S1.pdf]

Supplementary Table S1. Multicollinearity analysis of biometric-related traits in females.

Statistics/Parameters	Tolerance (1 – R^2)	VIF
Comb length	0.2048	4.8821
Wattles length	0.2085	4.7959
Nails color - White	0.2160	4.6306
Nails color - Courneous	0.2297	4.3534
Body weight	0.2376	4.2082
Wattles width	0.2464	4.0593
Comb width	0.2815	3.5524
Ornithological measurement	0.2916	3.4294
Back length	0.3067	3.2600
Longitudinal diameter	0.3359	2.9766
Nails color - Slate	0.3454	2.8950
Ocular length	0.3483	2.8708
Breast circumference	0.3715	2.6916
Ocular index	0.3798	2.6330
Keel of esternum length	0.4141	2.4149
Thigh length	0.4378	2.2841
Folding wing length	0.4627	2.1610
Nails color - Black/Courneous	0.4899	2.0413
Peak width	0.4973	2.0108
Ear lobes length	0.5090	1.9647
Tail length	0.5441	1.8381
Ear lobes width	0.5490	1.8215
Peak length	0.6767	1.4777
Neck length	0.7105	1.4075
Skull index	0.7746	1.2910
Presence/absence of spurs	0.7913	1.2637
Number of spikes in comb	0.8174	1.2234
Tarsus index	0.8748	1.1431
Nails color - Black/white	0.9089	1.1002

Interpretation thumb rule: VIF = 1 (Not correlated); 1 < VIF < 5 (Moderately correlated); VIF ≥ 5 (Highly correlated).
