# Supplementary material for: Discriminant Canonical Tool for Differential Biometric Characterization of Multivariety Endangered Hen Breeds
Source: Animals (Basel). 2021 Jul 26;11(8):2211. doi: 10.3390/ani11082211 (PMC8388411; doi:10.3390/ani11082211)
Supplement: Supplementary file 1 [file animals-11-02211-s001.zip › Supplementary Table S2.pdf]

Supplementary Table S2. Multicollinearity analysis of biometric-related traits in males.

Interpretation thumb rule: VIF = 1 (Not correlated); 1 < VIF < 5 (Moderately correlated); VIF ≥ 5 (Highly correlated).

Statistics/Parameters	Tolerance (1 – R^2)	VIF
Comb length	0.2237	4.4707
Wingspan	0.2270	4.4059
Anteroposterior tarsus diameter	0.2598	3.8492
Body weight	0.2866	3.4894
Peak color - White	0.2955	3.3841
Back length	0.3065	3.2631
Wattles length	0.3197	3.1276
Comb width	0.3267	3.0613
Ear lobes length	0.3403	2.9384
Ocular index	0.3406	2.9361
Peak color - Black	0.3634	2.7518
Longitudinal diameter	0.3842	2.6027
Peak width	0.3982	2.5115
Folding wing length	0.4102	2.4377
Thigh length	0.4105	2.4359
Peak color - Black/corneous	0.4111	2.4328
Ocular length	0.4118	2.4286
Breast circumference	0.4764	2.0990
Keel of esternum length	0.4896	2.0423
Ear lobes width	0.4981	2.0078
Ornitological measurements	0.5341	1.8722
Tarsus index	0.5843	1.7113
Peak color - Caramel/courneous	0.6274	1.5939
Skull index	0.6297	1.5882
Peak length	0.6874	1.4547
Neck length	0.6937	1.4415
Peak color – Black/white	0.6967	1.4353
Number of spikes in comb	0.7008	1.4269
