# Supplementary material for: Discriminant Canonical Tool for Differential Biometric Characterization of Multivariety Endangered Hen Breeds
Source: Animals (Basel). 2021 Jul 26;11(8):2211. doi: 10.3390/ani11082211 (PMC8388411; doi:10.3390/ani11082211)
Supplement: Supplementary file 1 [file animals-11-02211-s001.zip › Supplementary Table S3.pdf]

Supplementary Table S3. Appropriately classified females into their groups.

from \ to	White Sureña	Splash Sureña	Blue Sureña	Franciscan Sureña	Black Sureña	Partridge Sureña	White Utrerana	Franciscan Utrerana	Black Utrerana	Partrige Utrerana	Total	% correct
White Sureña	17	6	0	3	1	0	0	0	0	0	27	62.96%
Splash Sureña	7	15	2	3	3	0	0	2	0	0	32	46.88%
Blue Sureña	1	0	14	4	6	6	0	0	1	0	32	43.75%
Franciscan Sureña	3	3	1	27	0	0	1	0	0	0	35	77.14%
Black Sureña	1	5	5	1	49	9	0	0	0	0	70	70.00%
Partridge Sureña	3	1	4	1	4	32	0	0	0	0	45	71.11%
White Utrerana	1	0	0	0	0	0	14	15	0	0	30	46.67%
Franciscan Utrerana	0	0	0	0	0	0	5	56	0	0	61	91.80%
Black Utrerana	0	0	0	0	0	0	0	0	56	18	74	75.68%
Partrige Utrerana	0	0	0	0	0	0	0	0	7	59	66	89.39%
**Total**	33	30	26	39	63	47	20	73	64	77	472	71.82%
