# Supplementary material for: Discriminant Canonical Tool for Differential Biometric Characterization of Multivariety Endangered Hen Breeds
Source: Animals (Basel). 2021 Jul 26;11(8):2211. doi: 10.3390/ani11082211 (PMC8388411; doi:10.3390/ani11082211)
Supplement: Supplementary file 1 [file animals-11-02211-s001.zip › Supplementary Table S4.pdf]

Supplementary Table S4. Appropriately classified males into their groups.

from \ to	White Sureña	Splash Sureña	Blue Sureña	Franciscan Sureña	Black Sureña	Partridge Sureña	White Utrerana	Franciscan Utrerana	Black Utrerana	Partrige Utrerana	Total	% correct
**White Sureña**	11	0	0	0	0	0	0	0	0	0	11	100.00%
**Splash Sureña**	0	4	0	0	0	2	0	0	0	0	6	66.67%
**Blue Sureña**	0	0	4	0	2	0	0	0	0	0	6	66.67%
**Franciscan Sureña**	1	0	0	7	1	2	0	0	0	0	11	63.64%
**Black Sureña**	0	0	0	1	19	3	0	0	0	0	23	82.61%
**Partridge Sureña**	1	0	2	1	5	14	0	0	0	0	23	60.87%
**White Utrerana**	0	0	0	0	0	0	13	1	0	0	14	92.86%
**Franciscan Utrerana**	0	0	0	0	0	0	2	12	0	0	14	85.71%
**Black Utrerana**	0	0	0	0	0	0	0	0	13	1	14	92.86%
**Partrige Utrerana**	0	0	0	0	0	0	0	0	0	13	13	100.00%
**Total**	13	4	6	9	27	21	15	13	13	14	135	81.48%
