# Supplementary material for: Discriminant Canonical Tool for Differential Biometric Characterization of Multivariety Endangered Hen Breeds
Source: Animals (Basel). 2021 Jul 26;11(8):2211. doi: 10.3390/ani11082211 (PMC8388411; doi:10.3390/ani11082211)
Supplement: Supplementary file 1 [file animals-11-02211-s001.zip › Supplementary Table S5.pdf]

Supplementary Table S5. Leave-one-out cross-validation of females into their genotypes.

from \ to	White Sureña	Splash Sureña	Blue Sureña	Franciscan Sureña	Black Sureña	Partridge Sureña	White Utrerana	Franciscan Utrerana	Black Utrerana	Partrige Utrerana	Total	% correct
**White Sureña**	12	10	0	4	1	0	0	0	0	0	27	44.44%
**Splash Sureña**	10	11	2	4	3	0	0	2	0	0	32	34.38%
**Blue Sureña**	1	0	10	4	8	8	0	0	1	0	32	31.25%
**Franciscan Sureña**	6	6	1	21	0	0	1	0	0	0	35	60.00%
**Black Sureña**	1	5	9	3	40	12	0	0	0	0	70	57.14%
**Partridge Sureña**	3	1	6	1	8	25	0	0	0	1	45	55.56%
**White Utrerana**	1	0	0	0	0	0	7	22	0	0	30	23.33%
**Franciscan Utrerana**	0	0	0	0	0	0	11	50	0	0	61	81.97%
**Black Utrerana**	0	0	0	0	0	0	0	0	51	23	74	68.92%
**Partrige Utrerana**	1	0	0	0	0	0	0	0	9	56	66	84.85%
**Total**	35	33	28	37	60	45	19	74	61	80	472	59.96%
