# Supplementary material for: Discriminant Canonical Tool for Differential Biometric Characterization of Multivariety Endangered Hen Breeds
Source: Animals (Basel). 2021 Jul 26;11(8):2211. doi: 10.3390/ani11082211 (PMC8388411; doi:10.3390/ani11082211)
Supplement: Supplementary file 1 [file animals-11-02211-s001.zip › Supplementary Table S6.pdf]

Supplementary Table S6. Leave-one-out cross-validation of males into their genotypes.

from \ to	White Sureña	Splash Sureña	Blue Sureña	Franciscan Sureña	Black Sureña	Partridge Sureña	White Utrerana	Franciscan Utrerana	Black Utrerana	Partrige Utrerana	Total	% correct
**White Sureña**	5	2	0	2	0	1	0	1	0	0	11	45.45%
**Splash Sureña**	1	0	0	3	0	2	0	0	0	0	6	0.00%
**Blue Sureña**	0	0	0	0	2	4	0	0	0	0	6	0.00%
**Franciscan Sureña**	3	1	0	4	1	2	0	0	0	0	11	36.36%
**Black Sureña**	0	0	2	2	13	6	0	0	0	0	23	56.52%
**Partridge Sureña**	2	3	5	3	7	3	0	0	0	0	23	13.04%
**White Utrerana**	0	0	0	0	0	0	11	3	0	0	14	78.57%
**Franciscan Utrerana**	0	1	0	0	0	0	7	6	0	0	14	42.86%
**Black Utrerana**	0	0	0	0	0	0	0	0	12	2	14	85.71%
**Partrige Utrerana**	0	0	0	0	0	0	0	0	0	13	13	100.00%
**Total**	11	7	7	14	23	18	18	10	12	15	135	49.63%
