# Supplementary figures and images for: Discriminant Canonical Tool for Differential Biometric Characterization of Multivariety Endangered Hen Breeds
Source: Animals (Basel). 2021 Jul 26;11(8):2211. doi: 10.3390/ani11082211 (PMC8388411; doi:10.3390/ani11082211)

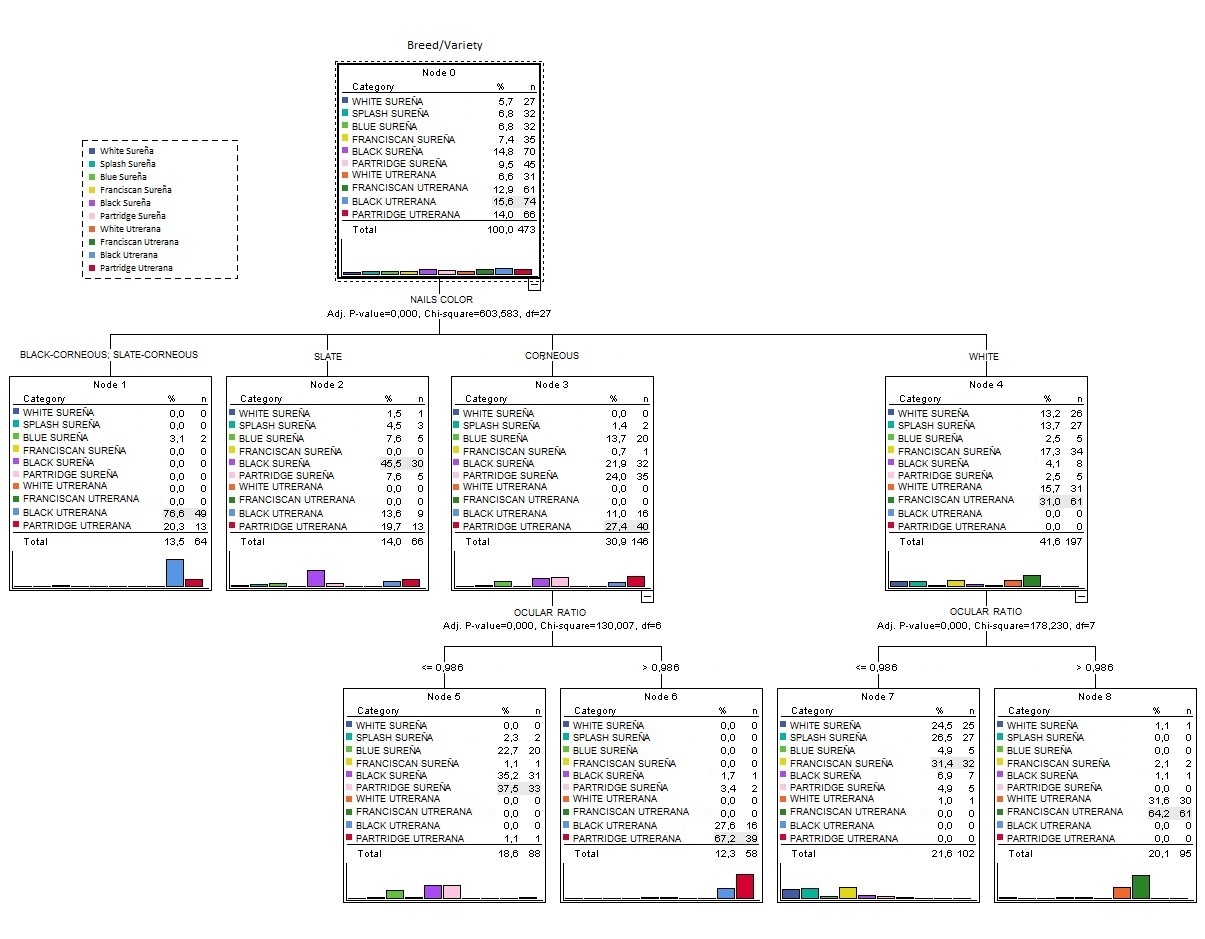

Supplement: Supplementary file 1 [file animals-11-02211-s001.zip › Supplementary Figure S1.jpg]

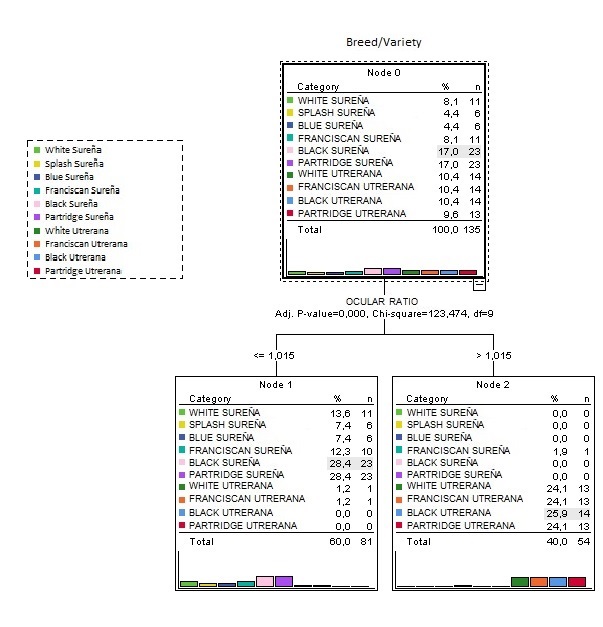

Supplement: Supplementary file 1 [file animals-11-02211-s001.zip › Supplementary Figure S2.jpg]
